# Supplementary material for: Evaluation of the Submaximal Treadmill-Based Fitness Test in Six Brachycephalic Breeds—A Follow-Up Study
Source: Animals (Basel). 2023 Nov 3;13(21):3413. doi: 10.3390/ani13213413 (PMC10648995; doi:10.3390/ani13213413)
Supplement: Supplementary file 1 [file animals-13-03413-s001.zip › animals-2627813-supplementary.pdf]

Supplementary Materials:

File S1. Descriptive statistics of demographic data of included dogs, divided into breed groups.

	Total (n = 72)	Boston Terriers (n = 28)	French Bulldogs (n = 32)	Small Brachycephalic Breeds (n = 12)
**Gender**				
Female	45	18	23	4
Male	27	10	9	8
**Age (years)**				
Mean (± SD)	4.2 ± 2.1	4.8 ± 1.9	3.7 ± 2.1	4.3 ± 2.3
**Weight (kg)**				
Mean (± SD)	9.8 ± 3.3	8.4 ± 1.6	12.7 ± 1.8	5.2 ± 1.9
**BCS (1-9)**				
Median (Range)	5 (4 - 8)	5 (4 - 6)	6 (4 - 8)	5 (4 - 7)
**Speed (km/h)**				
Mean (± SD)	4.9 ± 0.6	5.4 ± 0.5	4.7 ± 0.5	4.3 ± 0.6

File S2. Breathing noises (BN) of all dogs subdivided into breed groups at rest and after 5, 10 and 17 minutes of exercise.

			**Boston Terriers**		
**Time Point**		**At Rest**	**5 min**	**11 min**	**17 min**
**Number of subjects**		28	28	28	28
**No BN**		27/28 (96%)	22/28 (79%)	22/28 (79%)	21/28 (75%)
		**BN audible without stethoscope (n)**			
**Intermittent**	**Mild**	0	0	1	3
	**Moderate**	0	0	0	0
	**Severe**	0	0	0	0
**Constant**	**Mild**	0	1	1	0
	**Moderate**	0	0	0	0
	**Severe**	0	0	0	0
		**BN audible only with stethoscope (n)**			
**Intermittent**	**Mild**	1	3	3	3
	**Moderate**	0	0	0	0
	**Severe**	0	0	0	0
**Constant**	**Mild**	0	1	1	1
	**Moderate**	0	0	0	0
	**Severe**	0	0	0	0
			**French Bulldogs**		
**Time Point**		**At Rest**	**5 min**	**11 min**	**17 min**
**Number of subjects**		32	32	32	32
**No BN**		16/32 (50%)	14/32 (44%)	10/32 (31%)	12/32 (38%)
		**BN audible without stethoscope (n)**			

Intermittent	Mild	2	0	3	3
	Moderate	1	2	0	0
	Severe	0	0	0	0
Constant	Mild	1	3	4	5
	Moderate	6	4	6	5
	Severe	2	4	4	5
		**BN audible only with stethoscope (n)**			
Intermittent	Mild	2	0	3	2
	Moderate	0	1	0	0
	Severe	0	0	0	0
Constant	Mild	1	2	1	0
	Moderate	1	2	1	0
	Severe	0	0	0	0
		**Small Brachycephalic Breeds**			
**Time Point**		**At Rest**	**5 min**	**11 min**	**17 min**
**Number of subjects**		12	12	12	12
**No BN**		10/12 (83%)	7/12 (58%)	5/12 (42%)	6/12 (50%)
		**BN audible without stethoscope (n)**			
Intermittent	Mild	2	3	3	4
	Moderate	0	0	1	0
	Severe	0	0	0	0
Constant	Mild	0	0	0	0
	Moderate	0	0	0	1
	Severe	0	0	0	0
		**BN audible only with stethoscope (n)**			
Intermittent	Mild	0	2	3	1
	Moderate	0	0	0	0
	Severe	0	0	0	0
Constant	Mild	0	0	0	0
	Moderate	0	0	0	0
	Severe	0	0	0	0
